# Milrinone Relaxes Pulmonary Veins in Guinea Pigs and Humans

**DOI:** 10.1371/journal.pone.0087685

**Published:** 2014-01-31

**Authors:** Annette D. Rieg, Said Suleiman, Alberto Perez-Bouza, Till Braunschweig, Jan W. Spillner, Thomas Schröder, Eva Verjans, Gereon Schälte, Rolf Rossaint, Stefan Uhlig, Christian Martin

**Affiliations:** 1 Institute of Pharmacology and Toxicology, Medical Faculty of Rhenish-Westphalian Technical University Aachen, Aachen, Germany; 2 Department of Anesthesiology, Medical Faculty of Rhenish-Westphalian Technical University Aachen, Aachen, Germany; 3 Institute of Pathology, Medical Faculty of Rhenish-Westphalian Technical University Aachen, Aachen, Germany; 4 Institute of Pathology, Medical Faculty of Rhenish Friedrich-Wilhelms University Bonn, Bonn, Germany; 5 Department of Cardiac and Thorax Surgery, Medical Faculty of Rhenish-Westphalian Technical University Aachen, Aachen, Germany; 6 Department of Surgery, Luisenhospital Aachen, Aachen, Germany; 7 Department of Pediatrics, Medical Faculty of Rhenish-Westphalian Technical University Aachen, Aachen, Germany; University of Pittsburgh School of Medicine, United States of America

## Abstract

**Introduction:**

The phosphodiesterase-III inhibitor milrinone improves ventricular contractility, relaxes pulmonary arteries and reduces right ventricular afterload. Thus, it is used to treat heart failure and pulmonary hypertension (PH). However, its action on pulmonary veins (PVs) is not defined, although particularly PH due to left heart disease primarily affects the pulmonary venous bed. We examined milrinone-induced relaxation in PVs from guinea pigs (GPs) and humans.

**Material and Methods:**

Precision-cut lung slices (PCLS) were prepared from GPs or from patients undergoing lobectomy. Milrinone-induced relaxation was studied by videomicroscopy in naïve PVs and in PVs pre-constricted with the ET_A_-receptor agonist BP0104. Baseline luminal area was defined as 100%. Intracellular cAMP was measured by ELISA and milrinone-induced changes of segmental vascular resistances were studied in the GP isolated perfused lung (IPL).

**Results:**

In the IPL (GP), milrinone (10 µM) lowered the postcapillary resistance of pre-constricted vessels. In PCLS (GP), milrinone relaxed naïve and pre-constricted PVs (120%) and this relaxation was attenuated by inhibition of protein kinase G (KT 5823), adenyl cyclase (SQ 22536) and protein kinase A (KT 5720), but not by inhibition of NO-synthesis (L-NAME). In addition, milrinone-induced relaxation was dependent on the activation of K_ATP_-, BK_Ca_^2+^- and K_v_-channels. Human PVs also relaxed to milrinone (121%), however only if pre-constricted.

**Discussion:**

Milrinone relaxes PVs from GPs and humans. In GPs, milrinone-induced relaxation is based on K_ATP_-, BK_Ca_^2+^- and K_v_-channel-activation and on cAMP/PKA/PKG. The relaxant properties of milrinone on PVs lead to reduced postcapillary resistance and hydrostatic pressures. Hence they alleviate pulmonary edema and suggest beneficial effects of milrinone in PH due to left heart disease.

## Introduction

During and immediately after cardiac surgery, heart failure frequently occurs and therapy aims at improved ventricular contractility, maintained coronary perfusion and balanced myocardial oxygen consumption to facilitate separation from cardiopulmonary bypass. If heart failure is complicated by pulmonary hypertension (PH), immediate relaxation of the pulmonary vascular system is essential to avoid additional injury of the right ventricle. PH due to left heart disease is the most common cause of PH [1]. It affects primarily the pulmonary venous bed [2] and is therefore also called pulmonary venous hypertension (PVH) or postcapillary PH [3]. This aspect plays a pivotal role in the therapy of PVH, as vasodilators that act predominantly in the pulmonary arterial bed may increase the pulmonary perfusion and thereby enhance hydrostatic pressure, pulmonary edema and total pulmonary vascular resistance (PVR) [1]. Hence, successful therapy of PVH should preferably relax the pulmonary venous bed. However, as yet specific options to treat PVH are not available and the current recommendations are limited to the symptomatic therapy of left heart disease [4].

The phosphodiesterase-III (PDE-III) inhibitor milrinone is recommended for the therapy of right heart failure and PH [5], as it acts positive inotropic and decreases right ventricular afterload [6], even if inhaled [7]. Since milrinone is introduced in the therapy of heart failure, its relaxant effects have been carefully studied in porcine and ovine pulmonary arteries (PAs) [8], [9], in PAs from guinea pigs (GPs) [10] and in hypertensive PAs [8], [11].

In contrast, very little is known about the effects of milrinone in pulmonary veins (PVs). In a canine pulmonary occlusion model, milrinone was shown to reduce the pulmonary arterial and venous resistance during hypoxic pulmonary vasoconstriction [12]; but the direct vascular effects on PVs and the underlying mechanisms were not studied. Yet, as pointed out above, relaxation of PVs plays a key role in the treatment of PVH and left heart disease, as it counteracts left ventricular volume overload and wall stress. In addition, the pulmonary venous system contributes up to 40% to PVR [13], and in some species such as cats may even exceed 40% [14]. Thus, there is every reason to believe that the pulmonary venous bed is as important as the pulmonary arterial bed for PVR, PH and heart failure.

To access the question, whether and how milrinone relaxes PVs, we studied the vascular effects of milrinone in a recently established model that allows studying PVs and PAs independent from each other, i.e. **precision-cut lung slices (PCLS)** [17]. This method does even permit to study human tissue [15], [16] and here we show for the first time that milrinone relaxes human PVs and PAs. However, because access to human tissue is limited, we did the majority of experiments in lung tissue from GPs, because this species resembles human lung tissue better than that of mice or rats [15], [16].In GPs, milrinone relaxed PVs comparable to human lungs and this relaxation was dependent on cAMP/PKA/PKG and on the activation of K_ATP_-, BK_Ca_^2+^- and K_v_-channels. In addition, to determine the effects of milrinone on segmental vascular resistances, we studied milrinone-induced relaxation in the isolated perfused lung (GP) and report that milrinone lowers the postcapillary resistance. In conclusion, our data suggest favourably effects of milrinone in PH due to left heart disease.

## Materials and Methods

### GPs’ and Human Lung Tissue

Female Dunkin Hartley GPs (400±50 g; 6–8 weeks old) were obtained from Charles River (Sulzfeld, Germany). All animal care and experimental procedures were performed according to the rules of the Directive 2010/63/EU of the European Parliament. They were approved by the Landesamt für Natur, Umwelt und Verbraucherschutz Nordrhein-Westfalen (approval-ID: 8.87–51.05.20.10.245). Human PCLS were prepared from patients undergoing lobectomy due to lung cancer. After pathological inspection, cancer free tissue from a peripheral part of the lung was used. None of the patients showed any sign of PH (echocardiographic evaluation, histology) or took the sulfonyl urea glibenclamide as an antidiabetic drug. The study was approved by the ethics committee (EK 61/09) of the Medical Faculty Aachen, Rhenish-Westphalian Technical University (RWTH) Aachen. All patients gave written informed consent.

### Precision-cut Lung Slices (PCLS)

**Figure 1:**
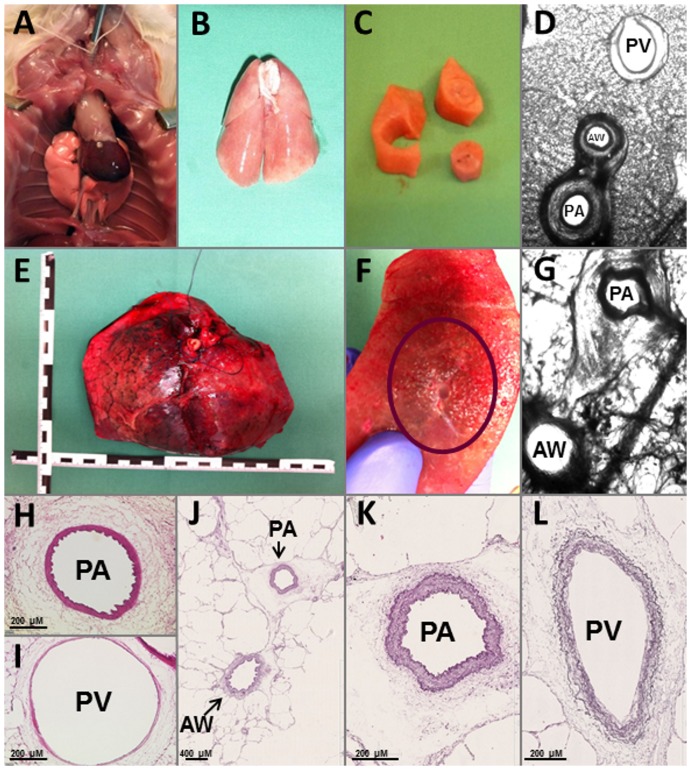
Preparation of PCLS from GPs and humans and their histology. A) GP: Tracheotomy; B) GP: lung filled with agarose; C) tissue cores; D) GP: PCLS during videomicroscopy; E) human: lung lobe filled with agarose; F) human: human lung tissue with airway and pulmonary artery; G) human: PCLS during videomicroscopy; H) GP: pulmonary artery (PA) with thick media and thrinkeld inner lining; I) GP: pulmonary vein (PV) with thin media; J) human: pulmonary artery (PA) and airway (AW); K) human: pulmonary artery (PA) with internal and external elastic lamina (diameter: 354 µm); L) human: pulmonary vein (PV) (diameter: 362 µm in width and 782 in height).

GPs received intraperitoneal anaesthesia with 95 mg kg^−1^ pentobarbital (Narcoren; Garbsen, Germany), which was verified by missing reflexes. Thereafter, PCLS (GP: n = 20; human lungs: n = 6) were prepared as described before [15], [17]. Whole lungs from GP (Fig. 1A/B) or human lung lobes (Fig. 1 E/F) were filled via the trachea respectively a main bronchus with 1.5% low-melting agarose and cooled on ice. Tissue cores were prepared (Fig. 1 C) and cut into 300 µm thick slices with a tissue slicer (Alabama Research & Development, Munford, AL, USA). PCLS were incubated at 37°C.

### cAMP Enzyme Immunoassay

To analyse cAMP, PAs/PVs were isolated out of tissue cores of agarose filled human lungs (n = 8) guided by the anatomical landmarks (see below). PAs/PVs were incubated in medium and after 30 minutes frozen by liquid N_2_. Cyclic AMP was quantified with ELISA-kits following the manufacturer’s protocol. For stabilization, all samples or standards were acetylated. To measure cAMP, all samples were diluted 1∶2 with 0.1 M HCL. The ELISA was evaluated at 405 nM (GENIOS, Tecan, Switzerland).

### Identification of the Vessels, Histology

Pulmonary vessels from GPs or humans were identified by their anatomical landmarks. PAs accompany the airways (Fig. 1D/G/J) and PVs lie aside. After the experiments, the identification of the vessels was additionally confirmed by histology. PCLS from GPs were stained with hematoxylin-eosin, where PAs show a wrinkled inner lining and a thick media (Fig. 1H) [17]; human pulmonary vessels were stained with elastica van Gieson, where PAs show an internal and external elastic lamina (Fig. 1K), in contrast to PVs which show only an external elastic lamina (Fig. 1L).

### Measurements and Imaging

**Figure 2:**
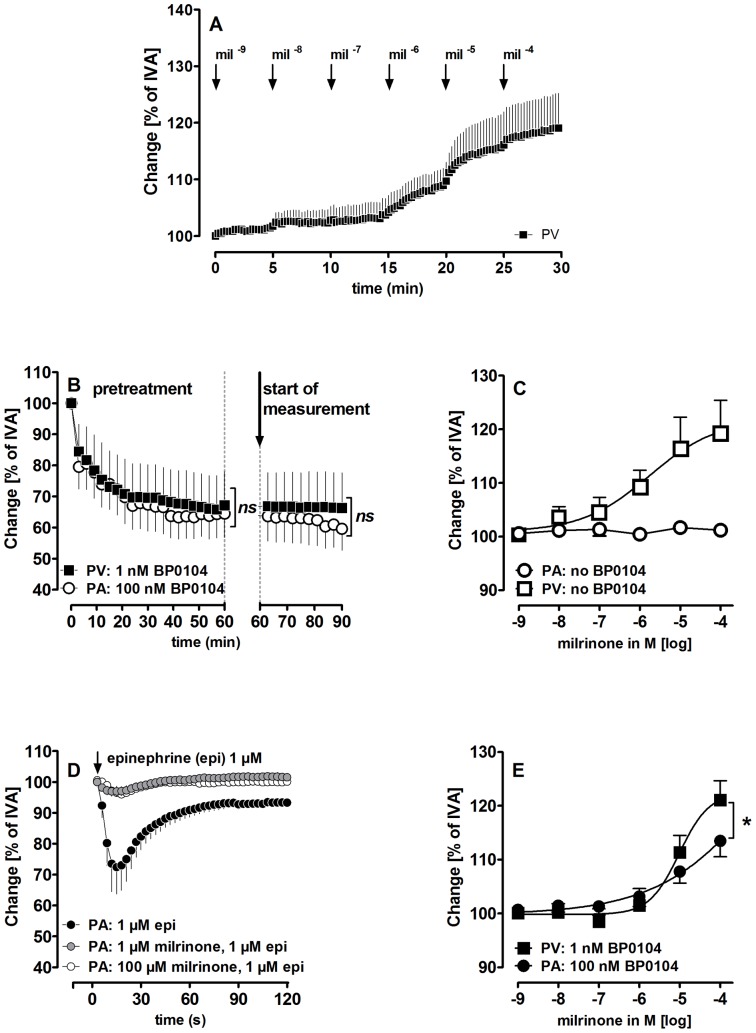
Vascular effects of milrinone in PAs and PVs with and without BP0104-induced pre-constriction. **A)** Kinetics of milrinone-induced relaxation in naïve PVs **B)** (**○**) PA: 100 nM BP0104 (n = 5); (▪) PV: 1 nM BP0104 (n = 5). The dashed line indicates the end of the pre-treatment and the start of the measurement. **C)** Milrinone in PAs/PVs without pre-constriction: (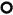) PA: milrinone (n = 12); (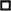) PV: milrinone (n = 8). **D)** Milrinone affects the contractile effect of epinephrine in PAs: (•) epinephrine (1 µM) (n = 5); (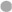) milrinone (1 µM), epinephrine (1 µM) (n = 3); (○) milrinone (100 µM), epinephrine (1 µM) (n = 5). **E)** Milrinone in pre-constricted PAs/PVs: (•) PAs: BP0104 (100 nM), milrinone (n = 7); (▪) PVs: BP0104 (1 nM), milrinone (n = 7). **B)** Statistics was performed by a linear mixed model. **E)** Asterics indicate different EC_50_ values. P<0.05 are considered as significant: * p<0.05, ** p<0.01 and *** p<0.001.

PCLS were exposed 5 minutes to each concentration of milrinone (Fig. 2A). To induce pre-constriction, PCLS were pre-treated for 1h with the endothelin_A_-(ET_A_)-receptor agonist BP0104 (Fig. 2B). To block a signaling pathway, PCLS were pre-treated for 1 h with the specific inhibitor. If both were required, PCLS were exposed simultaneously to both. Before the measurements, the *initial vessel area* (IVA) was defined as 100% and any relaxant or contractile effect (BP0104 or inhibitors) was indicated as “Change [% of IVA]”. Hence, a vessel area <100% indicates a contractile effect and a vessel area >100% indicates a relaxant effect. To compare relaxation of pre-treated vessels, the vessel area was defined after pre-treatment again as 100%. Concentration-response curves of the vasodilators were expressed as “Change [% of IVA]”. PCLS were exposed to the drugs one and two days after preparation. Control experiments were performed on consecutive sections. Pulmonary vessels were imaged and digitised (Fig. 1D/G, Leica Viscam 1280 or Leica DFC 280). The images were analysed with Optimas 6.5 (Media Cybernetics, Bothell, WA).

### Isolated Perfused Lung (IPL) of GP

Cavine lungs (n = 20) were prepared as described [18]. Briefly, intraperitoneal anaesthesia was performed (pentobarbital: 95 mg kg^−1^) and verified by missing reflexes. The GP was bled, the trachea cannulated and the lung ventilated with positive pressure (70 breaths/min). The left ventricle’s apex was cut and cannulas were placed in the pulmonary artery (perfusion inflow) and in the left atrium (perfusion outflow). The lung was perfused at constant flow (12,5 ml/min) with Krebs-Henseleit buffer, containing 2% bovine serum albumin, 0.1% glucose, 0.3% HEPES and 50nM salbutamol to prevent bronchoconstriction [19]. The temperature of the perfusate was maintained at 37°C with a water bath and the pH was maintained between 7.35 and 7.45 by CO_2_. Heart and lungs were removed and transferred into a negative-pressure chamber. Every 5 minutes a deep breath was applied to prevent atelectasis. Tidal volume, compliance, resistance, pulmonal arterial pressure (P_PA_), left atrial pressure (P_LA_) and the flow were continuously monitored. As soon as respiratory and haemodynamic values were stable over 20 minutes, BP0104 (20 nM) was added to the recirculating perfusion buffer (total volume 200 ml) to enhance PVR [20]. Ten minutes after the application of BP0104, milrinone (10 µM) was added. Thereafter, changes of the capillary pressure (P_cap_) were measured every 10 minutes by the double occlusion method [18]. The precapillary resistance (R_pre_) and postcapillary resistance (R_post_) were calculated by the following equations: R_pre_ = (P_PA_-P_cap_)/flow and R_post_ = (P_cap_-P_LA_)/flow.

### Agents and Culture Medium

All agents were bought from Tocris Bioscience (Ellisville, Missouri, USA), except milrinone and N-nitro-L-arginine methyl ester (L-NAME) which were obtained from Sigma-Aldrich (Steinheim, Germany) and BP0104 which was from BIOTRENDS (Wangen, Switzerland). All inhibitors are listed in Table 1.

**Table 1:** Overview of all used inhibitors.

inhibitor	target	IC_50_	used dosage
glibenclamide	K_ATP_-channels	20–200 nM	10 µM
iberiotoxin	BK_Ca_^2+^-channels	10 nM	100 nM
4-aminopyridine	K_v_-channels	0.3–1.1 mM	5 mM
SQ 22536	adenyl cyclase	1.4–200 µM	100 µM
KT 5720	PKA	60 nM	1 µM
L-NAME	NOS	25 µM	100 µM
KT 5823	PKG	0.23 µM	2 µM

In general, we expect complete inhibition of the target at concentrations about 10 times above the IC_50_ value [38], [56].

### Statistics

**Figure 3:**
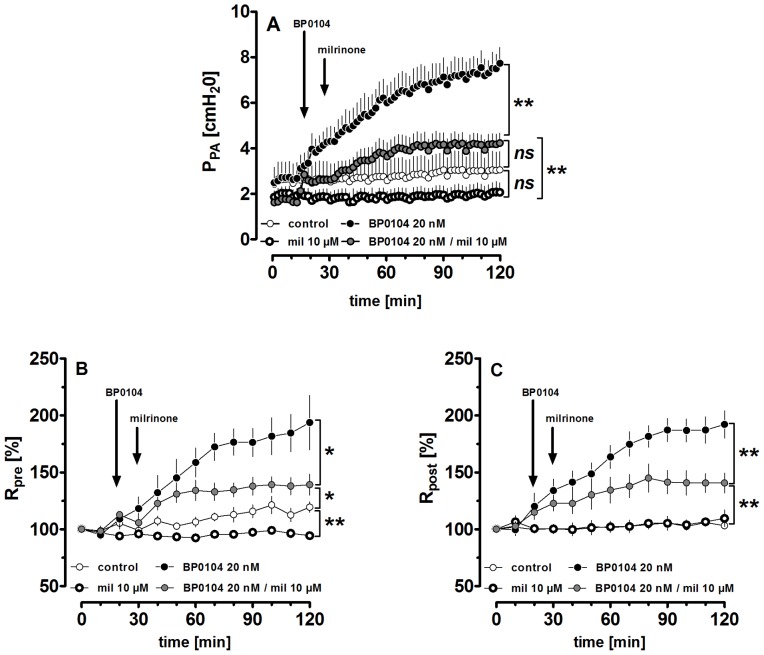
Vascular effects of milrinone in the GP isolated perfused lung. **A)** Effect of milrinone (mil) on the pulmonary arterial pressure (P_PA_) after and without pre-constriction with 20 nM BP0104: (○) control (n = 6); (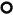) 10 µM mil (n = 3); (•) 20 nM BP0104 (n = 6); (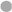) 20 nM BP0104, 10 µM mil (n = 5) **B)** Effect of milrinone on the precapillary resistance (R_pre_) after and without pre-constriction with 20 nM BP0104: (○) control (n = 6); (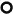) 10 µM mil (n = 3); (•) 20 nM BP0104 (n = 6); (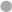) 20 nM BP0104, 10 µM mil (n = 5) **C)** Effect of milrinone on the postcapillary resistance (R_post_) after and without pre-constriction with 20 nM BP0104: (○) control (n = 6); (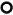) 10 µM mil (n = 3); (•) 20 nM BP0104 (n = 6); (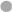) 20 nM BP0104, 10 µM mil (n = 5). (**A–C**) Statistics was performed by a linear mixed model. P<0.05 are considered as significant: * p<0.05, ** p<0.01 and *** p<0.001.

**Figure 4:**
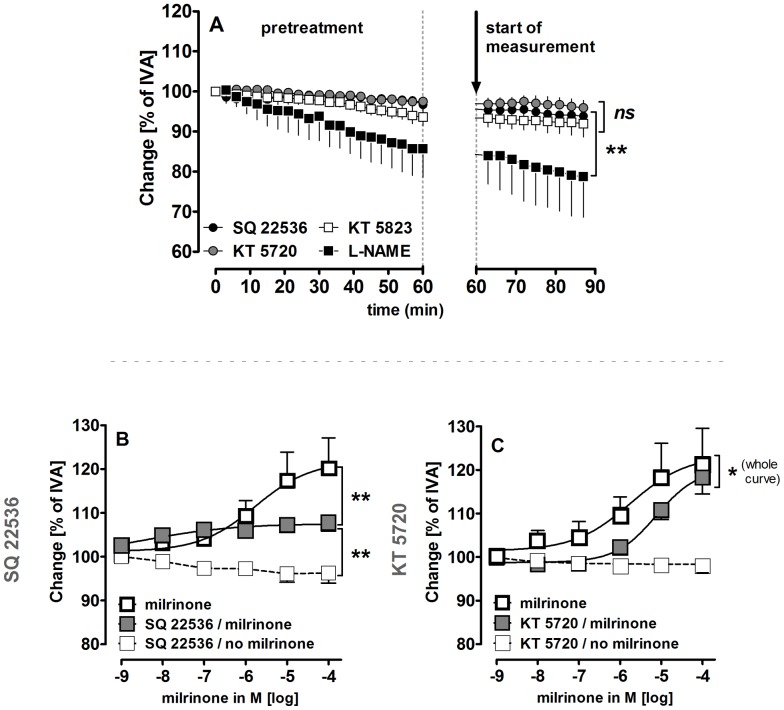
Influence of milrinone on cAMP/PKA-signaling in naïve PVs. **A)** (•) SQ 22536 (1 µM) (n = 4); (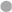) KT 5720 (1 µM) (n = 5); (□) KT 5823 (2 µM) (n = 5); (▪) L-NAME (100 µM) (n = 3); **B)** (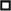) milrinone (n = 7); (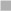) SQ 22536 (100 µM), milrinone (n = 7); (□) SQ 22536 (100 µM) (n = 4); **C)** (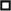) milrinone (n = 6); (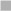) KT 5720 (1 µM), milrinone (n = 6); (□) KT 5720 (1 µM) (n = 6). **A)** Statistics was performed by a linear mixed model. **B–C)** Asterics indicate different EC_50_ values. P<0.05 are considered as significant: * p<0.05, ** p<0.01 and *** p<0.001.

Statistics was conducted using SAS 9.2 (SAS Institute, Cary, North Carolina, USA) and GraphPad Prism 5.01 (GraphPad, La Jolla, USA). The data in Fig. 2B, 3A–C, 4A, and 7B were analyzed by a linear mixed model analysis (SAS 9.2.). EC_50_ values were calculated using the standard 4-paramter logistic non-linear regression model (GraphPad). The AIC-criterion was used to select the most parsimonious model, i.e. a common bottom, top, slope and EC_50_ value in the regression model or the covariance matrix with the least number of parameters in the mixed model analysis (VC or AR(1)). Non-parametric analysis was performed by the Mann-Whitney U test. P-values were adjusted for multiple comparisons by the false discovery rate (FDR) and presented as mean+SEM or - SEM. P<0.05 was considered as significant and (n) indicates the numbers of animals.

## Results

In GPs, milrinone relaxed naïve PVs (119%), but not naïve PAs (Fig. 2C). To examine, if milrinone affects the contraction of naïve PAs, they were pre-treated with milrinone (1 µM and 100 µM) before being stimulated with epinephrine. Milrinone largely attenuated the epinephrine-induced contraction in naïve PAs (Fig. 2D). In PAs and PVs pre-constricted with the ET_A_-receptor agonist BP0104 (100 nM or 1 nM, respectively, Fig. 2B), milrinone relaxed PVs to 121% and PAs to 113% (Fig. 2E).

### Vascular Effects of Milrinone in the Isolated Perfused Lung (IPL) of the GP

To get insights whether milrinone-induced relaxation affects the PVR, we used the ex-vivo model of the IPL (GP). In comparison to control lungs, milrinone (10 µM) did not affect P_PA_ and R_post_ (Fig. 3A/C); but it prevented the slight increase of R_pre_ in control lungs (Fig. 3B). To mimic a characteristic of PH, the PVR was enhanced by the ET_A_-receptor agonist BP0104 (20 nM). This resulted in a remarkable increase of PVR, as indicated by the increase of P_PA_, R_pre_ and R_post_ (p<0.0001 for all; Fig. 3A–C). Thereafter, the perfusion of milrinone (10 µM) significantly lowered the BP0104-induced increase of PVR, which is demonstrated by the decrease of **P_PA_** (Fig. 3A), **R_pre_** (Fig. 3B) and **R_post_** (Fig. 3C).

### Role of cAMP/cGMP and NO in Milrinone-induced Relaxation

In order to investigate the relaxant mechanisms of the PDE-III inhibitor milrinone, PVs were pre-treated with the adenyl cyclase-inhibitor SQ 22536 (100 µM) or with the protein kinase A (PKA)-inhibitor KT 5720 (1 µM). Neither SQ 22536 nor KT 5720 altered the vascular tone of naïve PVs (Fig. 4A). Both however, reduced the relaxant effects of milrinone: Inhibition of adenyl cyclase attenuated the maximal relaxant effect of milrinone (Fig. 4B), whereas PKA-inhibition (Fig. 4C) caused a right-shift to higher EC_50_ values (21 µM versus 2.4 µM). However, neither SQ 22536 nor KT 5720 prevented the milrinone-induced relaxation completely.

**Figure 5:**
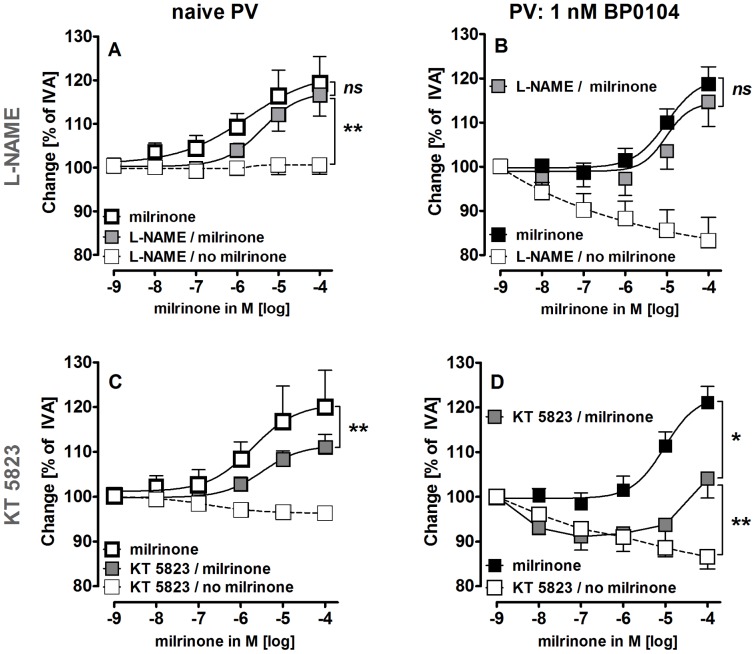
Influence of milrinone on cGMP/NO-signaling in PVs with and without pre-constriction. **A)** (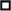) milrinone (n = 8); (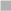) L-NAME (100 µM), milrinone (n = 8); (□) L-NAME (100 µM) (n = 3); **B)** (▪) BP0104 (1 nM), milrinone (n = 8); (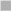) BP0104 (1 nM), L-NAME (100 µM), milrinone (n = 8); (□) BP0104 (1 nM), L-NAME (100 µM) (n = 8); **C)** (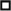) milrinone (n = 6); (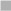) KT 5823 (2 µM), milrinone (n = 6); (□) KT 5823 (2 µM) (n = 6); **D)** (▪) BP0104 (1 nM), milrinone (n = 7); (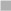) BP0104 (1 nM), KT 5823 (2 µM), milrinone (n = 7); (□) BP0104 (1 nM), KT 5823 (2 µM) (n = 6). **A–C)** Asterics indicate different EC_50_ values. **D)** Statistics was performed by the Mann-Whitney U test. P<0.05 are considered as significant: * p<0.05, ** p<0.01 and *** p<0.001.

We next studied the role of the NO/PKG-pathway in milrinone-induced relaxation. Inhibition of NO-synthesis by L-NAME (100 µM) increased the vascular tone of PVs (Fig. 4A), but it did not influence the relaxant effect of milrinone in naïve or pre-constricted PVs (Fig. 5A/B). In contrast, inhibition of protein kinase G (PKG) by KT 5823 (2 µM) significantly reduced the milrinone-induced relaxation in naïve and in pre-constricted PVs (Fig. 5C/D), indicating that the production of PKG is involved in milrinone-induced relaxation of PVs from GPs.

### Role of K^+^-channels in Milrinone-induced Vasorelaxation

**Figure 6:**
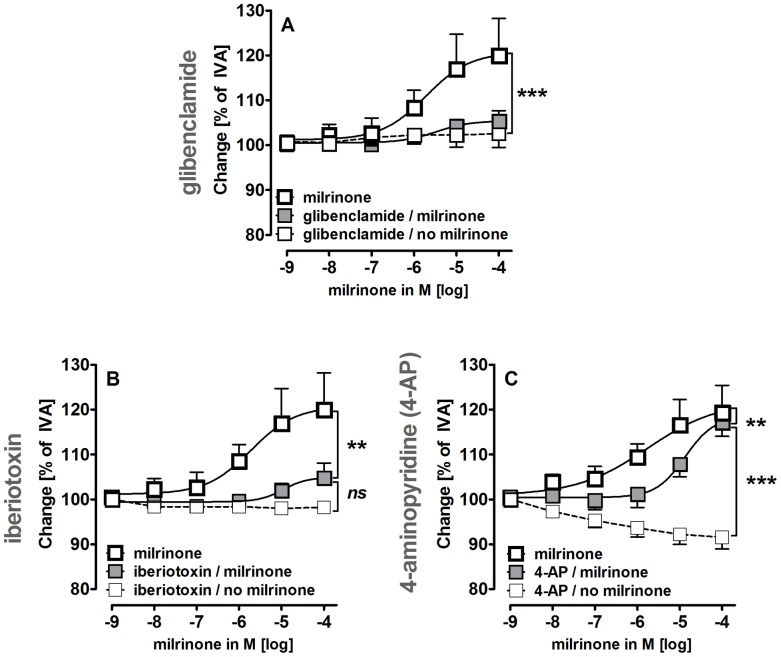
Impact of K^+^channels on milrinone-induced relaxation in PVs. **A)** (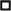) milrinone (n = 6); (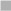) glibenclamide (10 µM), milrinone (n = 6); (□) glibenclamide (10 µM) (n = 4); **B)** (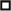) milrinone (n = 6); (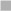) iberiotoxin (100 nM), milrinone (n = 6); (□) iberiotoxin (100 nM) (n = 3); **C)** (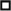) milrinone (n = 8); (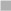) 4-AP (5 mM), milrinone (n = 8); (□) 4-AP (5 mM) (n = 8). **A–C)** Asterics indicate different EC_50_ values. **C)** The comparison of (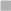) and (□) was performed by the Mann Whitney U test. P<0.05 are considered as significant: * p<0.05, ** p<0.01 and *** p<0.001.

To study the role of K^+^-channels in the milrinone-induced relaxation, PVs from GPs were pre-treated with the K_ATP_-channel inhibitor glibenclamide (10 µM), with the BK_Ca_^2+^-channel inhibitor iberiotoxin (100 nM) or with the K_v_-channel inhibitor 4-aminopyridine (4-AP; 5 mM). Glibenclamide and iberiotoxin alone did not alter the pulmonary venous tone, whereas 4-AP enhanced it (Fig. 6A–C). Inhibition of K_ATP_- and BK_Ca_^2+^-channels strongly reduced the relaxant effects milrinone (Fig. 6A/B). Inhibition of K_v_-channels did not attenuate the maximal relaxant effect in PVs, but provoked a right-shift to higher EC_50_ values (23 µM vs. 1.5 µM, Fig. 6C). In conclusion, the activation of all three K^+^-channels (K_ATP_ = BK_Ca_^2+^>K_v_) contributes to the relaxant effects of milrinone in GPs.

### The Relaxant Potency of Milrinone in Human Pulmonary Vessels

**Figure 7:**
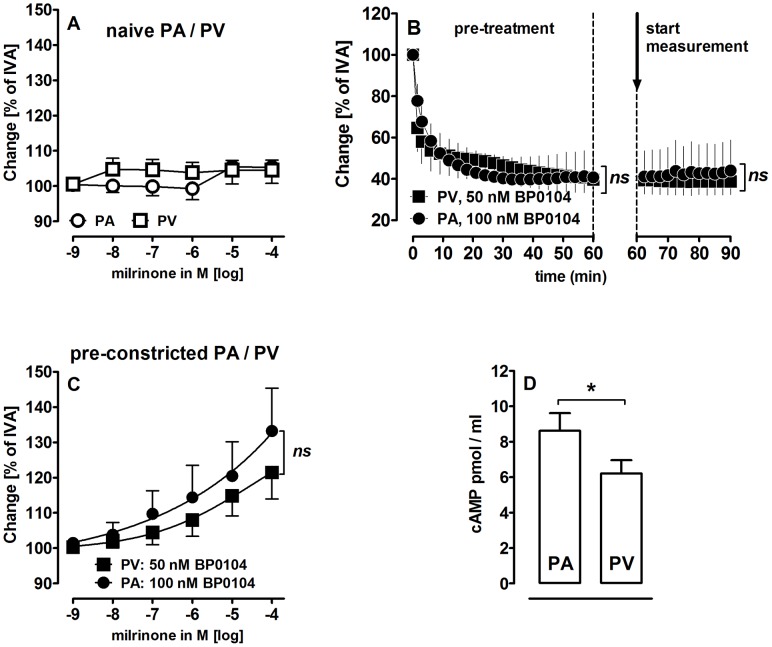
Relaxant potency of milrinone in human PAs and PVs. **A)** (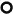) PA: milrinone (n = 4); (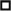) PV: milrinone (n = 3); **B)** (•) PA: BP0104 (100 nM) (n = 5); (▪) PV: BP0104 (50 nM) (n = 6); **C)** (•) PA: BP0104 (100 nM), milrinone (n = 5); (▪) PV: BP0104 (50 nM), milrinone (n = 5); **D)** Comparison of basal cAMP-levels of human PAs (n = 4) and human PVs (n = 8). (**B)** Statistics was performed by a linear mixed model. **C)** Asterics indicate different EC_50_ values. **D)** The comparison of PAs and PVs was performed by the Mann Whitney U test. P<0.05 are considered as significant: * p<0.05, ** p<0.01 and *** p<0.001.

Milrinone did not relax naïve human PAs and PVs (Fig. 7A). In order to pre-constrict human PAs and PVs equally, pulmonary vessels were pre-treated with various concentrations of the ET_A_-receptor agonist BP0104∶100 nM BP0104 constricted human PAs up to 44% of IVA and 50 nM BP0104 constricted human PVs up to 38% of IVA (Fig. 7B). In pre-constricted human pulmonary vessels, milrinone relaxed both PAs and PVs up to 133% and 121%, respectively (Fig. 7C). Pre-constricted human PAs relaxed significantly stronger to milrinone than those from GPs, in contrast pre-constricted PVs from both species relaxed equally. To analyse this different relaxant behaviour, we focused on cAMP-levels. We know that basal cAMP-levels from GPs are equal in PAs and PVs [21]. We now studied basal cAMP-levels in human PAs and PVs and found that they differ significantly (Fig. 7D).

## Discussion

Milrinone is widespread used in heart failure and PH. However so far, it is unknown if milrinone relaxes the pulmonary venous bed, the part of the pulmonary circulation which is mainly affected by PH due to left heart disease, the most common cause of PH [1]. Now we report that milrinone relaxes PVs (GPs/humans) and reduces the postcapillary resistance. We further show that milrinone-induced relaxation of PVs depends on cAMP/PKA/PKG, but is independent of NO. In addition, milrinone exerts relaxant effects via K_ATP_-, BK_Ca_^2+^-and K_v_-channel-activation. Notably, milrinone relaxes human PAs/PVs. This suggests that the present findings are relevant for the therapy of human cardiopulmonary diseases.

### Pre-constriction of GPs’ and Humans’ Pulmonary Vessels

We used GPs’ lungs of a non-disease model and human PAs/PVs from patients without PH. To mimic a characteristic of PH, pulmonary vessels (GP/humans) were pre-constricted with the ET_A_-receptor agonist BP0104, as endothelin plays a major role in PH and ET_A_-receptors are up-regulated [22]. In GPs, PAs and PVs were pre-constricted with 100 nM and 1 nM BP0104, respectively and both contracted equally up to 62% of IVA. Human PAs were pre-constricted with 100 nM BP0104 up to 44% of IVA, whereas human PVs were pre-constricted with 50 nM BP0104 up to 38% of IVA. Unlike PCLS, in the IPL the pulmonary arterial and venous bed cannot be separately treated with BP0104 and airways also constrict to BP0104. To prevent bronchoconstriction, a maximal concentration of 20 nM BP0104 was applied which constricted the pulmonary arterial and venous bed, as indicated by the increase of P_PA_, R_pre_ and R_post_ (Fig. 3A–C). Our results are in line with findings in porcine vessels [23] and once again exemplify the differences between the pulmonary arterial and venous bed, that are known with regard to adrenergic agents [17] endothelial NO-synthesis [24] or histamine and 5-HT [25].

### Milrinone Relaxes Pulmonary Veins in GPs’ and Humans’ Lung Tissue

Our data reveal that milrinone relaxes the pulmonary arterial and venous vascular bed: 1) In PCLS, milrinone relaxed naïve PVs, as well as pre-constricted PAs/PVs from central parts of the GP lung. 2) The value of this relaxation for PVR was confirmed in the IPL which allows determining the segmental vascular resistances of the lung by the double occlusion method; e.g. milrinone lowered the BP0104-induced increase of P_PA_, R_pre_ and R_post_. Reduction of R_post_ is of major clinical importance for PH due to left heart disease, as small PVs contributing to PVR also relax to milrinone. 3) Finally, the clinical relevance of milrinone-induced relaxation was confirmed in pre-constricted PAs/PVs from the peripheral human lung. - By the majority, milrinone-induced relaxation depends on pre-constriction; e.g. human PAs/PVs and GPs’ PAs. In contrast, GPs’ PVs relaxed unless being pre-constricted. On the other side, milrinone also affected the tone of naïve PAs, as it prevented the contractile effect of epinephrine. In a previous work [21] we showed that basal cAMP-levels are equal in naïve PAs/PVs from GPs and hence not responsible for the different relaxant response of PAs/PVs from GPs. Another idea suggests that GPs’ PVs dispose of a certain resting tone. This is supported by the fact that similar constriction of GPs’ PAs and PVs by BP0104 or U46619 (data not shown) requires lower concentrations in PVs than in PAs. We addressed this issue in the past [21] and illustrated that Ca^2+^-sensitization contributes to maintain this resting tone. However, this resting tone appears to be not applicable for the entire pulmonary venous bed of the GP. This is concluded from our experiments with the IPL, where milrinone only reduced R_post_ after the increase of the PVR by BP0104. Finally, this means that smaller PVs do not dispose of a resting tone, but require pre-constriction to relax to milrinone. Probably, these differences might be explained by the distinct behaviour of the differential segments of the pulmonary venous bed, as it was shown for the endothelial NO-release of the feline pulmonary arterial bed [26]. Further differences exist also between pulmonary vessels from GPs and humans; e.g. pre-constricted human PAs/PVs relaxed comparably to milrinone, but human PAs relaxed stronger than those from GPs. Aside higher basal cAMP-levels in human PAs versus PVs, the different degrees of pre-constriction of PAs/PVs from both species might also contribute to this observation. In comparison: human PAs were stronger pre-constricted than those from GPs. However, PVs from humans and GPs did not differ in their relaxant response to milrinone, although pre-constriction was different in both species. Next to possible interspecies differences, one should consider that GPs’ pulmonary vessels were obtained from the central part of the lung, whereas human pulmonary vessels were derived from the periphery. Hence, different vessels sizes were studied. This issue might also explain the distinct behaviour of GPs’ and humans’ PAs to milrinone, as was shown for various contractile and relaxant stimuli in pulmonary vessels of sheep [27].

### Mechanisms of Milrinone-induced Pulmonary Venous Relaxation

Milrinone relaxed pulmonary vessels from humans and GPs. Due to the limited access to human tissue and because lung tissue from GPs has been shown to mimic human tissue well [15], [16], we investigated the relaxant mechanisms of milrinone in GPs’ PVs. Milrinone relaxed naïve PVs by the activation of cAMP and PKA. This is in line with its action as a PDE-III-inhibitor and with the ubiquitous expression of PDE-III in the heart and lung [28]. These findings are also consistent with results from pulmonary or mesenteric arteries [8], [29]. Somewhat surprisingly, in PVs with and without pre-constriction, PKG-production also contributed to the relaxant effect of milrinone, although inhibition of NO-synthesis was without impact. Similar findings were reported by van der Zypp [30] and Kauffman [31] who found that PKG and cGMP contributed to milrinone-induced relaxation in rat aortas, even if the endothelium was denuded. Therefore we propose that the milrinone-induced PKG-generation might be based on a cross-talk between cAMP/PKA- and cGMP/PKG-signaling, which occurs on different levels [32]–[35]. This is corroborated by the observation that KT 5720 was less effective than SQ 22536 in reducing the relaxant effect of milrinone and suggests the production of other relaxant mediators downstream of cAMP such as PKG. In a previous work [21] we demonstrated that the Ca^2+^-sensitizer levosimendan, which exhibits also PDE-III-inhibiting properties, enhances intracellular cGMP and relaxes PKG-dependent pulmonary vessels, although inhibition of NO-synthesis by L-NAME or inhibition of guanylyl cyclase by ODQ did not influence levosimendan-induced relaxation. In addition, at high concentrations, milrinone inhibits also PDE-V, and further, PDE-III does hydrolyse not only cAMP but also cGMP. Both mechanisms might contribute to the role of PKG in milrinone-induced relaxation [36].

The second messengers cAMP and cGMP together with their downstream kinases PKA and PKG are involved in various intracellular processes leading to the relaxation of vascular smooth muscle cells; e.g. cAMP/PKA promote relaxation by activation of myosin light chain phosphatase (MLCP) [37] and by inhibition of myosin light chain kinase (MLCK) [34], whereas PKG activates MLCP [34]. Further, cAMP/PKA and cGMP/PKG stimulate potassium channels [38] leading to cell membrane hyperpolarisation and to reduced cytosolic Ca^2+^-influx via voltage-operated Ca^2+^-channels (VOCC) [39]. As a consequence, low cytosolic Ca^2+^ levels promote vasorelaxation by prevention of MLCK-activation [38]. It is therefore important that the pulmonary venous relaxant effects of milrinone appear to be mediated by the activation of potassium channels, namely K_ATP_-, BK_Ca_^2+^- and K_v_-channels. The three primary K^+^-channels appear to be of differing significance, because blocking of K_ATP_- and BK_Ca_^2+^-channels strongly attenuated milrinone-induced relaxation, whereas blocking of K_v_-channels provoked a right-shift of the concentration-response curve to higher EC_50_ values. Of note, in particular K_v_-channels are known to play a dominant role in the regulation of the pulmonary venous tone [21], [40]. Investigations in hypertensive pulmonary arteries confirmed the impact of BK_Ca_^2+^-channels for milrinone-induced relaxation [41], whereas in another study with non-pulmonary vessels, K^+^-channels did not contribute to the relaxant effect of milrinone [29]. Finally, our results do not only indicate the relevance of K^+^-channels for milrinone-induced pulmonary venous relaxation, but as well demonstrate important differences between pulmonary and systemic vessels.

Milrinone-induced relaxation was already shown for systemic veins [42]. However, results from the systemic circulation can’t be transferred to the pulmonary circulation in general, as both vascular beds express a remarkable distinct behaviour concerning the regulation of vascular permeability [43], the response to hypoxia [44], to cardiovascular agents [45], [46], to arachidonic acid [47], as well as differences in the NO-pathway [48], [49]. Therefore, questions concerning the pulmonary circulation can only be solved by examining pulmonary vessels themselves. Kato et al. [12] studied the relaxant effects of milrinone in the pulmonary vascular bed of the dog using a pulmonary arterial occlusion model. As the access to the pulmonary venous system is quite difficult, pulmonary venous pressures were calculated indirectly from the pulmonary capillary wedge pressure (PCWP). However, the PCWP reflects the left atrial pressure [50] or the pressure in large PVs [51], but not in small PVs, which are aside small pulmonary arteries mainly responsible for an increase in pulmonary vascular resistance [13], [52]. Isolated vessel preparations, isolated perfused lung models or the investigation of PVs in precision-cut lung slices represent a more appropriate approach to study the response of small PVs.

In this study, milrinone effectively relaxed human small PVs at concentrations of 1 µM which are consistent with plasma levels of 0.97–1.6 µM in men [53], [54]. Human and GPs’ pulmonary vessels were derived from healthy lungs, not from those with PH. To mimic the pathology of PH, pulmonary vessels of both species were pre-constricted with the ET_A_-receptor agonist BP0104. In some experiments NO-synthesis was also inhibited (L-NAME), to model another feature of PH [55]. In the IPL, we showed that milrinone effectively lowers the postcapillary resistance. From our studies we conclude that pulmonary venous relaxation likely contributes to the successful use of milrinone in PH associated to left heart disease by reducing the pulmonary venous tone, left-ventricular wall stress and pulmonary edema. In addition, milrinone might be a therapeutical option in pulmonary venous occlusive disease, a subgroup of PH, which primarily affects the pulmonary venous system.
