# Correction: Milrinone Relaxes Pulmonary Veins in Guinea Pigs and Humans

**DOI:** 10.1371/journal.pone.0097207

**Published:** 2014-05-02

**Authors:** The PLOS ONE Staff

Figure 1A is incorrect. It shows a tracheotomy procedure in a rat lung, not a guinea pig lung. The authors have provided a corrected version here showing a tracheotomy procedure in a guinea pig lung.

**Figure 1:**
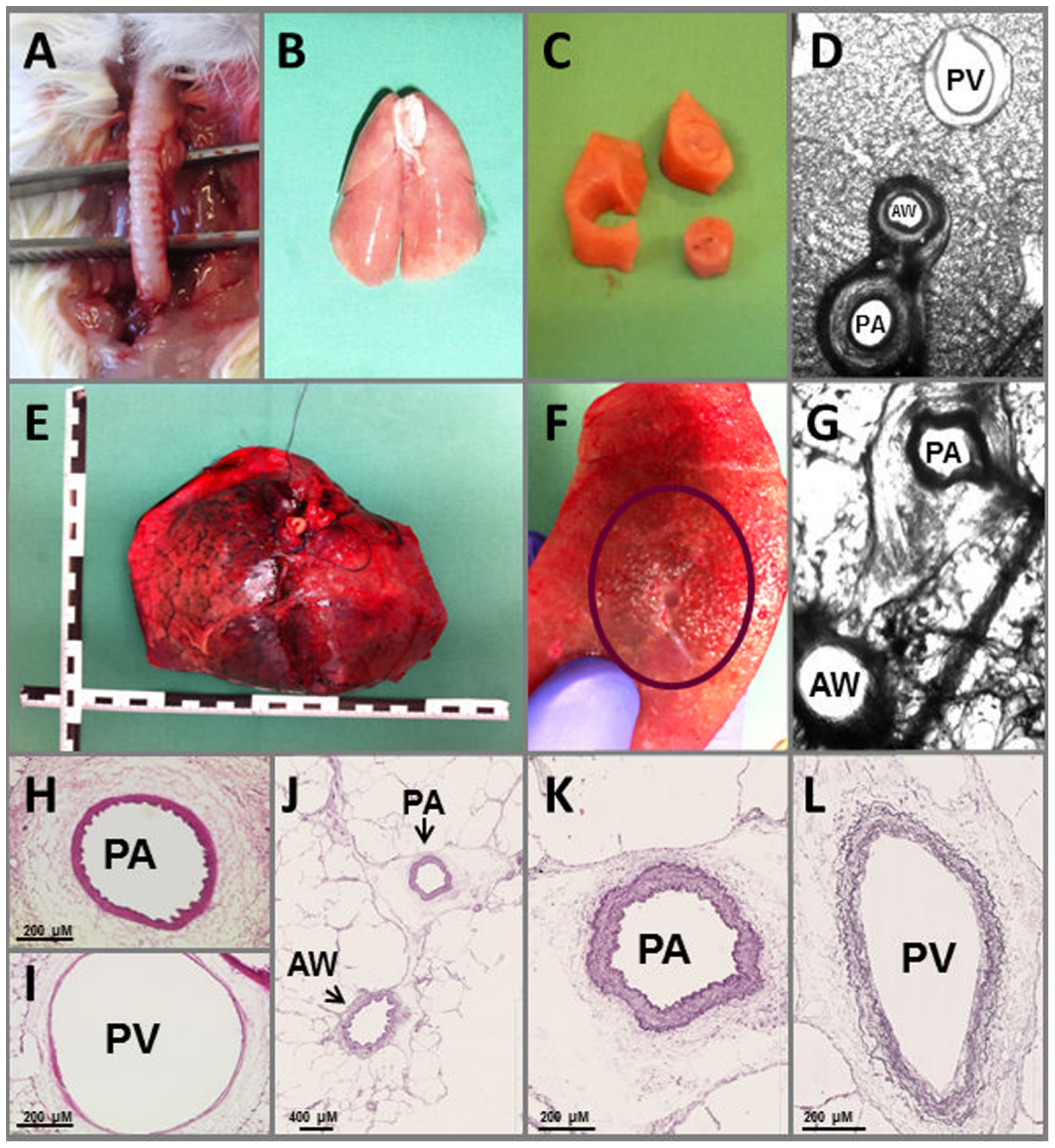
Preparation of PCLS from GPs and humans and their histology. A) GP**:** Tracheotomy; B) GP**:** lung filled with agarose; C) tissue cores; D) GP: PCLS during videomicroscopy; E) human: lung lobe filled with agarose; F) human: human lung tissue with airway and pulmonary artery; G) human: PCLS during videomicroscopy; H) GP**:** pulmonary artery (PA) with thick media and thrinkeld inner lining; I) GP: pulmonary vein (PV) with thin media**;** J) human**:** pulmonary artery (PA) and airway (AW); K) human: pulmonary artery (PA) with internal and external elastic lamina (diameter: 354 µm); L) human**:** pulmonary vein (PV) (diameter: 362 µm in width and 782 in height).
